# Automated, 3‐D and Sub‐Micron Accurate Ablation‐Volume Determination by Inverse Molding and X‐Ray Computed Tomography

**DOI:** 10.1002/advs.202200136

**Published:** 2022-05-06

**Authors:** Diego Monserrat Lopez, Valentine Grimaudo, Giulia Prone, Alexander Flisch, Andreas Riedo, Robert Zboray, Thomas Lüthi, Marcel Mayor, Martin Fussenegger, Peter Broekmann, Peter Wurz, Emanuel Lörtscher

**Affiliations:** ^1^ Science & Technology Department IBM Research Europe ‐ Zurich Säumerstrasse 4 Rüschlikon CH‐8803 Switzerland; ^2^ Department of Biosystems Science and Engineering ETH Zürich Mattenstrasse 26 Basel 4058 Switzerland; ^3^ Physics Institute Space Research & Planetary Sciences University of Bern Sidlerstrasse 5 Bern CH‐3012 Switzerland; ^4^ Department of Chemistry University of Basel St. Johanns‐Ring 19 Basel CH‐4056 Switzerland; ^5^ EMPA Swiss Federal Laboratories for Materials Science and Technology Überlandstrasse 129 Dübendorf CH‐8600 Switzerland; ^6^ Department of Chemistry Biochemistry and Pharmaceutical Science University of Bern Freiestrasse 3 Bern CH‐3012 Switzerland

**Keywords:** computed tomography, femto‐second laser pulses, laser ablation, mass spectrometry, molding, polydimethylsiloxane, X‐ray

## Abstract

Ablation of materials in combination with element‐specific analysis of the matter released is a widely used method to accurately determine a material's chemical composition. Among other methods, repetitive ablation using femto‐second pulsed laser systems provides excellent spatial resolution through its incremental removal of nanometer thick layers. The method can be combined with high‐resolution mass spectrometry, for example, laser ablation ionization mass spectrometry, to simultaneously analyze chemically the material released. With increasing depth of the volume ablated, however, secondary effects start to play an important role and the ablation geometry deviates substantially from the desired cylindrical shape. Consequently, primarily conical but sometimes even more complex, rather than cylindrical, craters are created. Their dimensions need to be analyzed to enable a direct correlation with the element‐specific analytical signals. Here, a post‐ablation analysis method is presented that combines generic polydimethylsiloxane‐based molding of craters with the volumetric reconstruction of the crater's inverse using X‐ray computed tomography. Automated analysis yields the full, sub‐micron accurate anatomy of the craters, thereby a scalable and generic method to better understand the fundamentals underlying ablation processes applicable to a wide range of materials. Furthermore, it may serve toward a more accurate determination of heterogeneous material's composition for a variety of applications without requiring time‐ and labor‐intensive analyses of individual craters.

## Graphical abstract

Laser ablation of materials combined with element‐specific analytics enables a direct correlation between the volumes ablated and its elemental composition. By crater molding by polydimethylsiloxane and X‐ray computed tomography thereof, a digital surface topology is created with sub‐micron accuracy. Large arrays comprising systematic ablation parameters variations enable volume, depth, and ablation rate to be determined in an efficient manner.

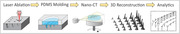

## 1 Introduction

Determining the chemical composition of a material is a general requirement in various areas ranging from the qualification of industrial production processes (e.g., in metalwork and ceramics industries) over the monitoring of material assemblies (e.g., semiconductor or photovoltaic industries) to the exploration of unknown matter (e.g., space science, geology). Thereby, the chemical analysis of homogeneously composed materials is somewhat easier compared to the one of heterogeneous ones, for example, layered or surface‐coated materials, as the homogeneous materials can be analyzed as a whole (e.g., by evaporation) or by small surface samples. For heterogeneous materials, various surface‐analytical methods exist, including optical, electrical, and electrochemical analytical methods, which can non‐destructively probe the surface from tens of nanometers to a few micrometers, mostly with a high lateral resolution to spatially map the surface. Examples include Raman scattering spectroscopy, X‐ray photon emission spectroscopy, and energy‐dispersive X‐ray spectroscopy. To retrieve information from material segments further away from the surface, where the penetration depth of radiation discontinues, additional, mostly destructive methods are employed that create access for aforementioned methods by removing hindering segments, for example by slicing, mechanical, or chemical mechanical polishing, (focused) ion‐beam milling etc. More elegant methods remove material while simultaneously determining the element composition to directly correlate it to the material released. Such methods include, for example, secondary ion mass spectrometry,^[^ ^1^, ^2^ ^]^ laser ablation inductively coupled plasma mass spectrometry,^[^ ^3^, ^4^, ^5^ ^]^ laser‐induced breakdown spectroscopy^[^ ^5^, ^6^ ^]^ or glow‐discharge mass spectrometry.^[^ ^5^, ^7^ ^]^ Meanwhile laser systems providing ultrashort pulse widths were even incorporated into TEMs to enable nanometer‐accurate ablation and in situ crystallographic surface analytics.^[^ ^8^ ^]^

**Figure 1:**
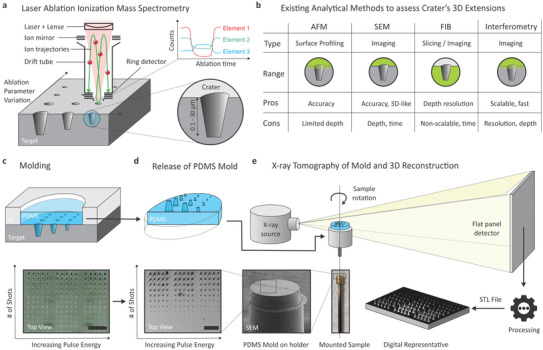
Schematics of laser ablation ionization mass spectrometry (LIMS) and approaches to analyze the ablation volumes: a) Laser ablation (LA) with a laser being focused on the sample to release material shot by shot. Subsequent chemical analysis by mass spectrometry. LA creates craters of different geometries and sizes that depend on ablation parameters such as laser polarization, wavelength, pulse energy, pulse width and number of pulses per position. b) While LIMS or similar methods achieve an excellent sensitivity and selectivity, the determination of the physical dimensions of ablation craters with depts exceeding 2–3 μm is much less precise as no scalable, single method exists to accurately and efficiently measure the corresponding dimensions in all three dimensions. Currently used methods include atomic force microscopy (AFM), scanning electron microscopy (SEM), focused ion beam (FIB) milling or white light optical interferometry, with advantages and disadvantages when applied to craters deeper than some μms as shown in the table. c) PDMS molding of the LA craters enables a fast, 3D, and nanometer‐accurate replication of the craters (the image on the bottom left shows an optical microscopy image) working up to full wafer scale covering the entire parametric LA variation (scale bar: 150 μm). d) The release of the mold from the sample wafer yields negatives of the cones protruding from the PDMS top surface that can better be characterized by, for example, SEM as depicted in the bottom image under a slight tilting of the PDMS sample (scale bar: 150 μm). e) Instead of SEM metrology, a scalable, 3D analysis of the cone's corpus is achieved by nanometric computed tomography (nano‐CT). Here, the PDMS mold is mounted into a 3D‐printed holder on a rod that is rotated around one single axis while X‐ray radiation emitted from a point source is recorded by a flat panel detector after passing through the PDMS sample. Typically, X‐ray images are acquired under 1440 different angles and data are reconstructed by dedicated software tools to yield a digital representation of the LA crater array.

One method from this class is laser ablation ionization mass spectrometry (LIMS), a measurement technique that allows for a direct investigation of the chemical composition of any solid sample at micrometer and nanometer scale, lateral and vertical, respectively, without the prerequisite of any sample preparation procedure. A pulsed laser source (here a femtosecond pulsed laser system that can provide laser irradiances at the 1 TW cm^−^^2^ level) is focused onto the solid's surface and the light–matter interaction causes photo‐ionization of the sample and removal—so called laser ablation (LA)—of a distinct layer thickness, which induces the formation of a plasma plume that expands normal to the sample surface. Ions released from the sample enter directly into a mass analyzer, without, for example, the need for any additional carrier gas as used in LA‐ICP‐MS. **Figure** **1**a shows a simplified illustration of such an apparatus (more details can be found under “Section 4”). The direct laser ablation and simultaneous analysis method of the matter released omits any sample preparation steps (thereby preventing sample‐preparation induced contamination) and is therefore independent of the chemical nature of the analytes to a certain degree. Due to the lack of any diluting steps (i.e., during sample preparation, e.g., dissolution or transportation, e.g., carrier gas), small sample amounts can be analyzed down to femtograms,^[^ ^9^ ^]^ resulting in high instrument detection sensitivities. Furthermore, element fractionation effects, originating from the transportation of ablated particles over long distances, as present in, for example, ICP‐MS, are non‐existent. However, the wide initial energy distribution of the ions generated by the laser limits the achievable mass resolution (*m*/Δ*m*) to below 800 for the LIMS instrument used in this study. The applied laser intensity and wavelength have to be optimized for the material investigated, otherwise the atomization and ionization efficiency might be limited.

While LA is widely established as depth‐profiling method,^[^ ^10^, ^11^, ^12^, ^13^ ^]^ non‐linear ablation profiles at micrometer scale craters represent an inherent challenge of the approach; with increasing depth, secondary effects start to play an important role and the ablation geometry starts to substantially deviate from the ideal cylindrical shape. Consequently, primarily conical (see Figure 1a), but sometimes even more complex‐shaped, rather than cylindrical crater geometries are created whose dimensions still need to be analyzed to enable a direct correlation with the element‐specific analytical signals. Various methods such as SEM,^[^ ^14^, ^15^, ^16^ ^]^ AFM,^[^ ^17^ ^]^ white light and laser interferometry,^[^ ^18^ ^]^ dark‐field and confocal microscopy^[^ ^19^ ^]^ have been employed to assess the crater's dimensions (see Figure 1b). Because of the high aspect ratio of craters with depths exceeding 2–3 μm, all these methods are somewhere limited—at least in one dimension—in the range or accuracy achievable, a scenario impaired by most materials optical opaqueness or surface roughness. An often used method to overcome the constrained access is to create cross‐sectional slices, a preparation step that is not only time‐ and labor‐intensive and therefore not scalable but may also induce contaminations (e.g., slurry residuals) or artefacts (e.g., mechanical forces).

We recently pioneered a method to replicate the crater's inner volumes (Figure 1c; more details under “Section 7”) into a nanometer‐accurate, polymeric negative.^[^ ^15^ ^]^ This versatile and scalable method working up to full wafer‐scale, enables an easier access to determine LA crater dimensions retrospectively, for example, through optical imaging, SEM etc. Replication molding of micro‐ and nanometer‐sized structures using polydimethylsiloxane (PDMS) is known to achieve sub‐10 nm accuracy through the conformal alignment of PDMS with surface‐modified materials (e.g., a 1–3 nm thick anti‐adhesive layer of silanes). The detailed and often numerous (in particular if the analysis comprises several dozens of craters in large LA studies with varying pulse width, energy, wavelength as well as number of pulses) analyses of the cones' dimensions (Figure 1c,d), however, remained labor‐intensive or were hindered for experimental reasons (e.g., cones being masked by adjacent ones, arrays not scattering enough, dense‐array loading effects etc.). For that reason, a scalable, high‐resolution method to efficiently analyze large arrays of 3D LA structures is strongly required.

With the discovery of X‐rays by Wilhelm C. Röntgen just 125 years ago,^[^ ^20^ ^]^ first radiographs were acquired, enabling a non‐destructive—a part from the ionizing radiation—imaging of the internal structure of materials. Driven by the German law that allowed claims for damage of defective products, technical radiography became more and more spread in the 20th century. Concurrently, the use of X‐rays was introduced into medicine. In 1912, the use of X‐rays for crystallographic investigations was first introduced by Max von Laue^[^ ^21^ ^]^ and later by William H. (father) and William L. Bragg (son). An important step was made in 1968 when Godfrey N. Hounsfield demonstrated the first CT scan of a pig brain (with a scan‐time of nine days).^[^ ^22^ ^]^ The reconstruction algorithms were based on the mathematical framework by Allen M. Cormac,^[^ ^23^ ^]^ while today's commercial CT algorithms use filtered back‐projection, often referred to as FDK‐algorithm.^[^ ^24^ ^]^ X‐rays generated by synchrotron radiation achieve even sub‐nanometric resolution with a high material contrast nowadays,^[^ ^25^ ^]^ applicable directly to aforementioned laser‐ablation studies.^[^ ^26^ ^]^ The drawbacks of using synchrotron radiation, however, are the limited availability and high costs of regularly performing X‐ray ptychography, not to mention the efforts for sample preparation and alignment. In our work, we chose much more accessible X‐ray computed‐tomography (nano‐CT) systems with lower resolution, and combined them with an automated analysis of PDMS‐molded craters to determine all relevant parameters at scale. In the following, we explain the principles underlying nano‐CT, systematically characterize the new metrology approach using a suitable test system based on bottom‐up fabricated LA‐mimicking structures, and finally showcase the applicability of the approach in a prototypical LA study conducted in a well‐studied silicon sample.

## 2 PDMS Molding and Imaging by Nano‐CT

X‐ray computed tomography (CT) is a non‐destructive imaging method based on computer‐assisted processing of multiple X‐ray projections of the specimen acquired under different angles. During data acquisition the specimen is rotated in the X‐ray beam in small angular steps. X‐ray projection images of each step are recorded by an X‐ray detector. The series of these X‐ray projections are used to reconstruct cross‐sectional images without the need to physically slice the specimen. In our commercial system (see “Section 7” for details), the X‐ray radiation is emitted from an X‐ray source with a very small focal spot size, which allows to achieve high spatial resolution. Within the collimator‐free cone‐shaped X‐ray beam the sample is rotated by 360°. The X‐ray radiation is attenuated in the specimen by material‐specific absorption, thereby creating a transmission‐path dependent intensity pattern on the flat panel detector (Figure 1e). To achieve the highest resolution (specified by the manufacturer to be below 500 nm) the sample has to be positioned as close to the X‐ray source as possible, allowing high magnification of the X‐ray projections on the flat‐panel detector. To achieve that, PDMS molds are mounted via 3D printed holders (Figure 1e) in ≈3 mm distance from the source after being cast inside a compartment (placed either over the entire LA crater array or on selected regions of interest; see Experimental Section). These samples with 1.5 mm diameter contain large LA studies and are imaged under 1440 different angles.

## 3 Data Processing Pipeline

**Figure 2:**
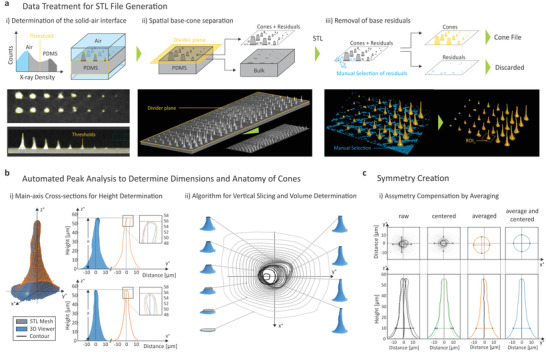
Basic data pipeline from raw nano‐CT data to Standard Triangulation and Tesselation Language (STL) surfaces and automated analytics thereof: a) Conceptual (upper row) and exemplary (lower row) X‐ray tomography data treatment for the creation of a STL cone surface file: i) Solid (PDMS)–air interface determination by selecting an appropriate threshold in the X‐ray density histogram (using local optimizations by software algorithms); this step yields the desired solid–air interface as indicated by the yellow boundaries. ii) Spatial separation of base/bulk and cone sections by a spatial divider plane and export of the determined surfaces of the cone sections into an STL file. iii) Removal of base/bulk residuals surrounding the cones by semi‐automated selection of connected features, yielding separated, individual cones in an array. This digital, inverted representation of the laser ablation craters is then stored into a single STL file. b) Automated peak analysis: i) Example of a high‐resolution STL surface mesh, consisting of triangles with areas smaller than 1 μm^2^ (left), that enables arbitrary viewing, for example, as cross‐sections along the vertical *z*‐axis (right panels). The base coordinate system is chosen with respect to the incident laser (in the *z*–*y* plane), causing typical asymmetries due to its finite angle of incidence. Algorithms are used to create contour profiles (right panel) that can be used to directly determine the height of the cones in respect to the bulk base. ii) An algorithm creating vertical slices at arbitrary heights above base and tunable spacing between the slices (e.g., 2 μm) enables volume determination by integration. c) Compensation of laser‐ablation intrinsic asymmetries by either centering or radially averaging (or combining both of them) of sliced areas along one common axis (e.g., defined by the base). This averaging or centering and averaging procedures do not change the resulting volume of the cones but enables, for example, the evolution of the laser ablation craters to be better visualized based on parameter variations within the arrays or algorithms to be executed faster on large arrays (see Figures 4 and 5).

The 3D data reconstructed from the X‐ray projections with the nano‐CT's proprietary software (see “Section 7”) is then treated such that the PDMS‐air interface is revealed, neglecting all the inner structures normally of interest in X‐ray studies (left panel of **Figure** **2**a). For that purpose, data is processed in a commercial CT analysis software (see “Section 7”) using its advanced surface determination approach for single material, yielding the interface as yellow surface (cross‐sectional views in lower left panel). Then, the PDMS bulk section, required to keep all cones together upon casting, is removed by a spatial divider plane (middle panel), yielding separated LA cones and a cuboid. This spatial separation should be done close to the bottom of the cones in order not to lose information (e.g., for small number of pulses or low energies). As the specimen's surface is not completely planar, this step consequently produces residuals around the cones. The surfaces of the LA cones including the residuals are converted to a mesh and exported as a standard triangulation and tesselation language (STL) file. Using the software Image‐ware (see “Section 7”), the residuals are removed in a semi‐automated manner (right panel) by an algorithm that considers connected structures as entities. After the data treatment, the individual cones only with pitches and levels as in the initial LA sample are merged and stored into a cone surface file.

STL files for individual cones are generated by selecting the region of interest (ROI) by the array pitch (panel (iii) in Figure 2a) and are processed in Python by importing them with the numpy‐stl library. This allows the data corresponding to all data points and surface normals of the triangulated mesh to be read‐out. From this package, different algorithms are run to calculate parameters such as height, surface area or volume, as well as to get a visual representation of regions of interest. For instance, the cone can be intersected with vertical planes along the cone's axes to obtain the exact vertical cross‐sections (Figure 2b(i)), or with horizontal planes to create slices to investigate the anatomy of the cones at arbitrary heights (Figure 2b(ii)).

Vertical cross‐sections cannot take into consideration the asymmetries of the cones with respect to the *z*‐axis, an effect caused by the non‐perfect‐orthogonal incidence of the ablation laser on the specimen's surface but potentially also by strain reliefs present inside the PDMS after extracting them from the high‐aspect ratio craters. The asymmetry, being inherent to the ablation process or not, can be compensated by averaging and centering each cross‐section to one common main axis creating a symmetric representation of a cone profile as shown in Figure 2c. Many equidistant horizontal slices are created, each one of them (with asymmetric circular shapes) being composed by a circumference line for which the center of gravity is computed. Each circumference can then be centered along the common main axis, as shown in Figure 2c, labeled as “centered”. Alternatively and to simplify subsequent analytical analysis, a single “radius” value can be computed by averaging the distance of each slice's circumference point to the center of gravity (Figure 2c, labeled as “averaged”). The symmetric representation of the cone profile is achieved by aligning all centers of gravity and plotting the radius value for each one of the slices at the corresponding height (Figure 2c, labeled “averaged and centered”). The horizontal slicing approach also enables volume determination by integrating the area of all the slices to be computed (summing the area of all slices multiplied by the distance between them). The total surface is calculated directly by summing up the areas of the triangulated mesh, while the total height is the distance from the furthest data point to the horizontal plane at the base.

## 4 Empirical Validation by Bottom–Up Test Structures

**Figure 3:**
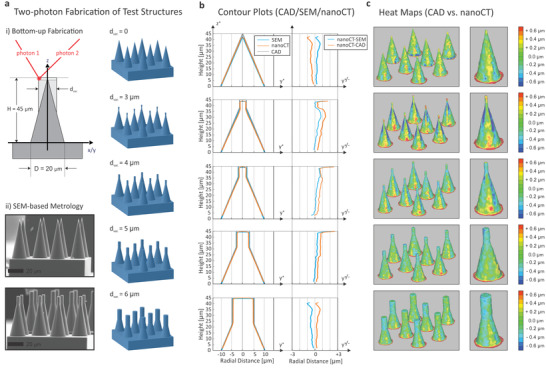
Empirical determination of the nano‐CT resolution by bottom–up fabricated, LA‐like test structures: a) Test structures fabricated bottom–up by two‐photon lithography. The cones aim at mimicking typical LA crater geometries and dimensions with a primary conical shape and an additional cylindrical extension on the top, with the latter having variable diameters, *d*_var_, of 0, 3, 4, 5, or 6 μm. The test structures area is upscaled to a 2 × 4 array as shown in the CAD design on the right and illustrated by SEM images for *d*_var_ = 0 and 3 μm on the bottom left for the fabricated structures (scale bar: 20 μm). b) Averaged cone contour plots derived by nano‐CT and compared to SEM and original CAD design files. The CAD design is corrected in height and diameter by SEM metrology to account for resist shrinkage upon cross‐linking. c) 3D heat maps show the difference between SEM‐corrected CAD and nano‐CT data all around the cone's surfaces. As shown both in (b) and (c), the absolute differences between SEM‐corrected CAD and nano‐CT data are less than 1.0 μm on the main cone barrel and around 1–2 μm for the cone's cylindrical extension where typically the upper most, spiky section at the top of the cones is not captured entirely by nano‐CT.

As LA craters can be geometrically quite complex,^[^ ^15^ ^]^ making an assessment of the spatial resolution difficult, we first validate and quantify the nano‐CT‐imaging approach of LA‐like polymeric structures by fabricating and characterizing bottom–up test structures. For that purpose, we employ a two‐photon process to polymerize a resist with sub‐micron resolution (see “Section 7”; smallest width of structures in the *X* *Y* plane: 160 nm; surface roughness: 20 nm, minimal layer thickness in *Z*: 100 nm) to create cone‐like specimens by bottom–up 3D printing (upper panel in **Figure** **3**a). Based on previous studies^[^ ^15^ ^]^, the test cones were designed to represent typical dimensions, for example, *D* = 20 μm for the bottom diameter and a height h = 45 μm. Additionally, to mimic the prominent top region of interest, we superimposed a cylinder with *d*_var_ = 3, 4, 5, and 6 μm in diameter, aligned with the top peak of the cone (illustrated by the light gray rectangles in the upper panel of Figure 3a). All five test geometries were multiplied to an array of eight identical cones each (right row of Figure 3a). A cross‐check using scanning electron microscopy confirmed that the structures were created as desired. However, with a slight minus allowance (*D* = (19.85 ± 0.25) μm; *h* = (44.85 ± 0.25) μm, and *d*_max_ = (9.90 ± 0.10) μm) most likely due to examination under high voltage and under vacuum (gray data in Figure 3b). This consistent size‐reduction was corrected in the CAD files for later comparison.

The test structures were then imaged by nano‐CT and the surfaces extracted as stereo lithography (STL) format file according to the data pipeline described above. Figure 3b depicts radial contour profiles for the desired cones (gray lines depict in the CAD data), the SEM derived ones (extrapolated from five measurement points at the beginning and end of the structures, as well as the cylinder starting points) and the nano‐CT profiles (average). The absolute deviation between nano‐CT and SEM is shown in the right row of Figure 3b and shows deviations lower than 1 μm radially and height inaccuracies of 1–4 μm. The STL format allowed for a numerical comparison with the CAD reference file used as input for the test structures. The deviation from the SEM‐corrected CAD file is plotted as a color‐coded heat map with a range from +0.7 μm (red) to −0.7 μm (blue) in Figure 3c. Overall, this analysis shows that the nano‐CT accurately images the test structures with voxel size and with an edge length around 300–500 nm on the specimen–air interface. Notably, this resolution is comparable to long‐range measurements performed by high‐resolution SEM over several μm where dimensions are determined in a cross‐sectional, tilted specimen–beam orientation. SEM characterization, however, requires full electro‐optical clearance (no masking by other structures under 80°–90° tilting) and a rotation of the sample to determine its full 3D shape in sequential, time‐ and labor‐intensive single‐step measurements. In contrast, the nano‐CT‐based analysis can be done over a large area with automated data processing to determine all LA‐relevant parameters (depth, area, volume, etc.) at scale and a resolution below 1 μm (or a voxel size of about 300 nm^3^).

## 5 Exemplary Laser‐Ablation Study in Silicon

After this empirical assessment of the novel approach for LA analysis purposes, we focus on real LA specimens and conduct a typical LA study with systematic parameter variation on a Si(100) sample, which is probably the most widely used material in literature. The laser pulse energy was varied step‐wise from 0.13 to 1.57 μJ and the total number of laser pulses between 1 and 10 000. To generate craters of various dimensions, 13 different laser burst counts (1, 2, 5, 10, 20, 50, 100, 200, 500, 1000, 2000, 5000 and 10 000 laser pulses) were applied for each of the applied pulse energies: (1.58 ± 0.07), (1.27 ± 0.06), (1.05 ± 0.05), (0.80 ± 0.05), (0.38 ± 0.04), and (0.13 ± 0.04) μJ. Each unique combination of burst count and pulse energy was applied to a fresh position on the sample with five repetitions per parameter set to allow for average building. The lateral distance between craters of different burst counts and between repetitions of the same burst count was set to 50 μm. The crater arrays created by the different pulse energies were separated by a pitch of 100 μm, resulting in a total area of 0.6 × 1.7 mm^2^, fitting well on a single PDMS mold to be analyzed in one nano‐CT run. For the analysis, only the shape and the dimensions of the craters created in Si will be discussed and no mass spectrometric element signals were recorded consequently.

**Figure 4:**
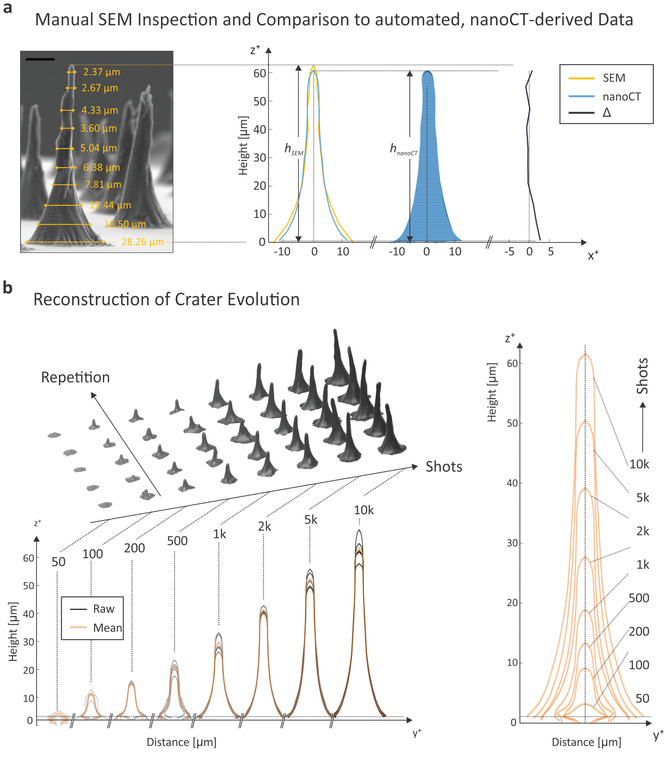
Direct comparison of conventional SEM metrology with nano‐CT Results and evolution of Si laser ablation craters: a) Discrete SEM cross‐sectional diameter measurements (Scale bar: 20 μm) were interpolated as indicated in the SEM micrograph (ignoring the tilting of the cone), while the nano‐CT profile was created as shown in Figure 3C for the corresponding peak (created by 10 000 shots of ≈1.58 μJ pulse energy). The blue filled curve is the cross‐section of the STL file along the respective orientation. On the right side, the differential plot between SEM measurements and automated nano‐CT is plotted. b) The digital representation of a typical LA study with 50–10 000 shots is shown for a fixed LA energy of ≈1.58 μJ. The profiles at the bottom show the statistics of all five repetitions with the mean values depicted as orange overlay. Based on the latter data, the evolution of LA craters can be constructed as displayed on the right.

As very small top sections of the PDMS mold are not recorded by nano‐CT, we first take control measurements by SEM to compare the height with the nano‐CT results. **Figure** **4**a shows the height and ten discrete diameter measurements by SEM taken to extrapolate a (symmetric) contour profile. As already noticed with the test structures in Figure 3b, around 1–4 μm of the spiky top segment are missing in the nano‐CT profile, irrelevant for the overall surface and volume determination. Apart from that, the profiles show a good agreement over the main part of the cone surface with some deviation in the bottom section. The relative contour lines are plotted on the right side. Processing the data for a full array and all repetitions, as shown in the upper 3D of Figure 4b, yields the contour profiles plotted in the bottom panel. The raw data in gray are overlayed with the average over all repetitions in transparent orange. This way, the evolution of the cones (or in reality the craters) can be plotted, for example for an increasing number of laser shots, as shown in the right panel. Starting from the smallest recognizable crater for the highest pulse energy, one can see a transformation of the crater shape from convex to concave. Furthermore, the sidewall angle to the sample surface increases with deeper layers of the craters. The aperture angle of the crater on the upper side of the walls becomes steeper with a larger number of applied laser pulses. Also, the crater diameter increases when a larger number of pulses hits the same sample location, but to a lesser extent than the crater depth.

**Figure 5:**
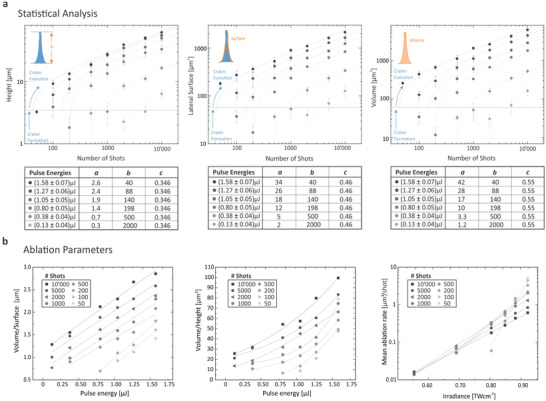
Automated determination of laser ablation parameters and statistical analysis thereof: a) Automated analysis of an entire LA study can be performed using the algorithms described, for example, to plot height, surface, or ablated volume, for an increasing number of shots (from 50 to 10 000) and an increasing pulse energy (from (0.13 ± 0.04) to (1.58 ± 0.07) μJ). The dotted lines represent power law curves manually constructed as a guide to the eye with the parameters depicted in the corresponding tables below. b) Determination of the volume‐to‐surface ratio, the volume‐to‐height ratio, and the mean ablation rate as a function of irradiance (for the same parameter variations as plotted in panel (a)).

In **Figure** **5**a, results are depicted for height, lateral surface area and volume as a function of shots and for various pulse energies in a log–log representation: below 100 shots, the small indentations at the beginning of the crater formation can only be recorded by nano‐CT when using the highest energy of (1.58 ± 0.07) μJ while being removed upon divider plane or residual cleaning for lower energies. For the smallest applied pulse energy (0.13 ± 0.04) μJ, at least 2000 shots are required to produce a detectable cone by nano‐CT and related processing algorithms. By plotting the height of the PDMS cones versus the applied number of laser shots in a double logarithmic representation (left panel), one can see an uniform trend for the different pulse energies. The data follow the power law function *y* = *a* × (*x* − *b*)^*c*^, with *a* being related to the pulse energy, *b* being the number of laser shots required to create an ablation crater and *c* being related to the ablation rate. The power law trends displayed in Figure 5a (dotted lines) were created by keeping *c* constant for each plot, and *b* for all the three plots while adjusting the parameter *a*. The parameters used are given in the tables in Figure 5a. Fitting the data to other models including the accumulation model^[^ ^27^ ^]^ yielded good agreements but the derived parameters are not directly comparable to literature due to different instrument settings, such as pulse length, wavelength, and environment at which laser ablation was performed. The power law behavior indicates that the ablation rate decreases with the number of laser shots for all tested pulse energies in the same way. The declining rate is described by the exponent of the formula. When considering the removed depth for the ablation rate, the declining rate is about 1/3 for Si material and for the applied pulse profile. Variable *b* describes the minimum number of laser shots that are required to overcome the initial crater formation step and to reach a crater that progresses with a constant geometry into the depth. This value may be affected by the measurement resolution of the nano‐CT and should be further investigated for smaller craters, applying a higher resolution measurement technique, for example direct measurement of the craters with interferometry. Variable *a* is laser power dependent. The smaller the pulse energy the smaller is the value. Knowing *b* and *c*, it is possible to predict the number of pulses needed to profile a certain layer thickness. This formula allows for a depth calibration during the ongoing depth profiling measurement. When considering the ablated volume instead of the ablated depth for the ablation rate (right panel), the exponent *c* becomes 1/2 for this material and the applied pulse profile. The exponent *c* and the variable *a* were adjusted by assuming the same variables *b*, as they describe the same crater formation step. Because the volume of a symmetric cone is calculated according to the formula *V* = 1/3 × *A* × *H*, where *A* is the base area and *H* is the height, *A* is expected to contribute less to *V* when considering the derived fit equations (exponent *c* would be about 0.2, assuming a similar trend for the evolution of the base area with an increasing number of laser pulses). In other words, with successive laser shots the cone volume grows mainly in height and to a lesser extend in width. As shown in the corresponding graphics, the minimal volume that can be detected are around 10–20 μm^3^. All curves plotted do not show a maximum in the ablation volume per pulse as expected from laser ablation theory of parabolic dipole ablation.^[^ ^28^ ^]^ To reach this point, increased laser irradiances would be required, a range which was not investigated using our instrument. Typically, the removal of small material layers per single laser shot are of interest in our studies. This approach allows high vertical resolution studies of the chemical composition of solids using our LIMS system.

Figure 5b provides additional information about ablation parameters such as the volume/surface ratio (left), the volume/height ratio (middle), or the ablation rate for an increasing number of shots (right). The volume/surface ratio follows a linear dependence on the pulse energy, with the same slope for craters created with more than 200 laser shots. This indicates that the shape of the cones increases about uniformly with increasing crater depth. The slow slope observed below 500 shots can be explained by an initial crater formation step. First, the flat surface of the substrate needs to be disrupted and a stable crater shape needs to be created. This step may involve non‐linear processes like the reflection of the light and the introduction of defects in the surface structure. Once the crater is set, it propagates with a constant shape of the craters. In line with the observation regarding the linear trends observed for the pulse energy versus volume/surface ratio, a quadratic behavior is observed for the middle panel of Figure 5b for the pulse energy versus volume/height ratio; the higher the applied pulse energy, the faster the volume increases as in comparison with the height. When plotting the logarithm of the mean ablation rate as derived from the ablated volume, as a function of the applied irradiance linear trends can be observed. For simplicity, the laser irradiance corresponds to applied pulse energy divided by the pulse width and measured ablation crater area. The slope of these functions slightly decreases with an increasing number of laser shots, which indicates a continuous loss of the pulse energy arriving at the bottom of the crater. A part of the pulse energy is not used for the removal of material, probably because it is involved in the formation of defects in the surrounding material or due to reflections at the surface. The more shots are averaged for the mean ablation rate, the larger the fraction of energy that is not used for the removal of material, resulting in less averaged ablated material. Also here, a significant change of the slope is observed for craters created with less than 500 shots, a fact which may again be explained by the initial crater formation step where the shape changes from concave to convex as discussed above. The laser ablation threshold for Si can be estimated based on the data displayed in Figure 5b, right panel (note that the *y*‐axis is shown in logarithmic scale). For the campaign of 5000 and 100 laser shots, a threshold at the level of about 0.5 and 0.8 TW cm^−2^, respectively, can be estimated, which is slightly below the literature value of about 2 TW cm^−2^.^[^ ^29^ ^]^ The study conducted with 5000 laser shots at the lowest applied pulse energy in this study can be compared with^[^ ^29^ ^]^ where small scale structures were observed on the Si sample after applying 60 000 laser shots at 0.4 0.5 TW cm^−2^. For full quantitative study of the laser ablation threshold of Si using our setup, however, single laser shot campaigns need to be conducted which is out of scope of this study here.

## 6 Conclusion

In conclusion, we presented a quantitative analytical method on laser ablation craters that combines PDMS molding and 3D imaging by X‐ray nano‐CT as well as automated data analysis. The generic approach reproduces all relevant parameters such as ablation volumes, ablation depth, ablation rate etc. with sub‐micron resolution as it was showcased by bottom–up test structures that mimic typical ablation geometries. The PDMS molding can be repeated multiple times, if needed, without detrimentally changing the sample in each step. Hence, even sequential studies are possible where the ablation process is continued after repeatedly creating intermediate PDMS molds of the craters. While the PDMS curing (24 h) and nano‐CT image acquisition (4–16 h) are generally time‐consuming but either not labor‐intensive or fully automated, the processing of the 3D topology can now be done in a few tens to hundreds of seconds due to its digital representation, an analysis time three to five orders of magnitude faster compared to other non‐scalable and non‐automatable characterization methods such as AFM or SEM. As a result, comprehensive datasets of a statistically sufficient number of craters can now be analyzed in detail such that the underlying ablation mechanisms can be elucidated as it was exemplarily shown for the archetype material silicon. With the ablated volumes now better determinable at scale, for example, in large ablation studies with systematic variation of the relevant parameters including pulse energy, number of pulses etc. the generic and material‐independent approach enables a more accurate material analysis when combined with elemental analysis. A part from the application in material analysis including semiconductor industry, geology, space research, etc. the molding and nano‐CT approach could also be useful for any other determination of surface topologies that are highlighted by a high surface roughness with difficult metrological access.

## 7 Experimental Section

### NanoScribe

A nanoscribe “photonic professional 2” instrument was used for the two‐photon polymerization process to create LA‐mimicking test structures.

### Ablation Laser

A femtosecond laser system that outputs pulses of about 190 fs at 1 kHz with a fundamental wavelength of 775 nm was used as ablation ion source. For this study, the fundamental beam was converted within the optical path of the beam, outside the laser system, to 258 nm using harmonic generators (STORC, Clark‐MXR Inc.). Note, the beam profile after harmonic generation was expected to be of lower Gaussian quality as the fundamental beam. Various optical elements, for example, dielectric mirrors, guided the converted beam toward the mass analyzer, which was located inside a vacuum chamber, at a base pressure of  10^−7^ mbar. Inside the chamber, the laser beam was focused by means of a doublet lens, which was fixed directly above the mass analyzer and just below the entrance window to the vacuum chamber, along the central axis of the mass analyzer, directed toward the sample surface. The latter was placed on a micro‐translation stage with the sample surface normal to the central axis of the mass spectrometer, and thus the irradiation axis. The sample surface was positioned at the focal point of the lens, which is typically situated about 0.5 mm below the entrance optics of the mass analyzer and 250 mm below the lens system. The irradiance was calculated by the measured pulse energy at sample surface (below the mass analyzer) divided by the pulse width and the measured laser ablation crater area.

### PDMS Molding

To perform PDMS molding, a customized mold was created for this particular purpose. Before starting the molding, the sample and the mold were coated with a silane monolayer. A fluorinated silane was used to keep the PDMS from sticking to the sample and, at the same time, to be able to reproduce the structures with high accuracy. After the silane incubation (30 min) under vacuum, the mold with the sample inside was kept in the oven for 1 h at 80 °C. It was then filled with PDMS, taking care to remove any potential bubbles. The filled mold was left open at room temperature for 1 h to allow the smaller bubbles to diffuse through the PDMS and then cured in the oven for 24 h at 60 °C.

### Computed Tomography

A 160 kV source (using a LaB_6_ electron emitter limited to 100 kV) on a commercial nano‐CT system, Easytom XL Ultra from RX Solutions, with a focal spot size significantly smaller than 1 μm was used in a collimator‐free cone‐beam geometry and a flat‐panel detector with 1920 × 1536 pixels with a size 127 × 127 μm^2^ each. The data acquisition was performed with 70 kV and roughly 150 μA, 1440 angular positions and an adequate geometrical magnification. A two‐step approach was applied: a global scan of the full sample diameter, followed by a local scan with higher resolution. The final voxel size was 0.5 μm. The software XAct, used for data acquisition and reconstruction, was a proprietary one from the CT scanner manufacturer RX Solutions. The advanced surface determination algorithm from the commercial CT analysis software VGStudioMax3.3 from Volumegraphics determined the material–air interface based on the local gray value differences, taking into account the neighboring voxels. As a starting contour for calculating the interface, a threshold was defined based on the histogram of the gray value distribution. For the definition of the background (air) and the material (PDMS) a representative region of interest was used to define the threshold. The determined surfaces were converted to a triangle mesh using the grid‐based (precise and watertight) algorithm by applying the option “create closed surface”. The software Imageware (Version 13.1) used for processing the STL data is a trademark of Siemens Lifecycle Management Software Inc.

### SEM Inspection

A Hitachi SEM with an acceleration voltage of 3–10 kV was used to image both the two‐photon polymerization test and the PDMS LA structures coated with 8–12 nm of Au to drain charges.

### STL Analysis

Numpy‐stl Python's library enabled to read with STL files, extracting the raw and unstructured 3D triangulated surfaces by the unit normal and vertices. The volume for each one of the peaks composing this sample could be determined by integrating the volumes of the triangular columns, defined by each one of the surfaces composing the STL file, and the plane defined as base of the sample. With access to the raw data of the STL files (vertices on the triangulated surface) any other parameter such as height, axis at given heights, or bottom area could be easily calculated, as well as any cross‐section of the structure.

### Statistical Analysis

For the study of bottom–up test structures, there was a sample size of *n* = 8 structures for each type (5 different structure types in total). No preprocessing of the data was done, and data was presented as mean ± s.d. For the laser ablation study in Silicon, 13 different laser burst counts were considered for six different pulse energies, a sample size of *n* = 5 repetitions for each combination were performed and studied. The combination for which less than three repetitions were distinguishable from the material surface in the STL file (very small volume ablated) were discarded from the study, what explains the absence of data points for low pulse energies and low shots. All data was presented and plotted as mean ± s.d. All STL data was created using the Imageware software (Version 13.1) as explained in computed tomography section, and all analysis was performed using Python's libraries as explained in the previous section STL analysis.

## Conflict of Interest

The authors declare no conflict of interest.

## Author Contributions

E.L. proposed the use of nano‐CT for LA analytics. LA studies were conducted by V.G.G.P. created the molds and made adaption to X‐ray imaging. A.R. optimized the LIMS setup for measurements. T.L. and R.Z. conducted X‐ray tomography. A.F. analyzed the X‐ray data. D.M.L. developed analytical software and also carried out the study of test structures. The results were discussed among all authors and all authors were involved in writing the manuscript. The project was supervised by P.B., P.W., and E.L.

## Data Availability

The data that support the findings of this study are available from the corresponding author upon reasonable request.
